# Prognostic impact of anemia on the mortality of United Arab Emirates nationals with cardiovascular disease

**DOI:** 10.5339/qmj.2022.3

**Published:** 2022-03-12

**Authors:** Saif Al-Shamsi, Ghada S M Al-Bluwi, Maitha Al Shamsi, Nouf Al Kaabi, Sara Al Khemeiri, Noura Baniyas

**Affiliations:** ^1^ Department of Internal Medicine, College of Medicine and Health Sciences, United Arab Emirates University, Al-Ain, United Arab Emirates E-mail: salshamsi@uaeu.ac.ae

**Keywords:** Anemia, cardiovascular disease, mortality, United Arab Emirates

## Abstract

Background: Cardiovascular disease is the leading cause of death worldwide. Multiple risk factors, including low hemoglobin levels, have been associated with poor outcomes in patients with cardiovascular disease. However, the long-term impact of anemia on death has not been investigated in high-risk patients in the United Arab Emirates. Therefore, this study evaluated whether anemia is a significant predictor of mortality in United Arab Emirates nationals with cardiovascular disease over 10 years.

Methods: A retrospective cohort study was conducted in an adult population of United Arab Emirates nationals with a history of cardiovascular disease, recruited from a tertiary healthcare facility. Electronic medical records between April 2008 and December 2008 were reviewed, and follow-up was conducted until December 2019. The survival functions for all-cause mortality in the presence and absence of anemia were compared using univariate Kaplan–Meier analysis with a log-rank test. The association between anemia and all-cause mortality was evaluated using a multivariable Cox regression model.

Results: A total of 224 patients were included in the follow-up for 10.5 years. At baseline, 46% of the patients had anemia, with a mean Hgb level of 105.5 ± 28.0 g/L. Patients with anemia were older (68 vs. 63 years, p = 0.001) and had a higher rate of chronic kidney disease (37.5% vs. 17.5%, p = 0.001) than those without anemia. A total of 77 (34.4%) deaths were recorded by the end of the follow-up period. Risk of all-cause mortality was significantly higher in patients with anemia than in those without (hazard ratio = 2.03, 95% confidence interval = 1.22–3.40, p = 0.006). Age and chronic kidney disease were also statistically significant predictors of death (p < 0.001 and p = 0.001, respectively).

Conclusion: Anemia is an independent predictor of all-cause mortality in United Arab Emirates nationals with underlying cardiovascular disease. Early intervention and treatment for anemia may improve clinical outcomes in this population.

## Introduction

Cardiovascular disease (CVD) is a major global public health problem, causing an estimated 17.9 million deaths each year worldwide.^1^ The identification of modifiable factors that affect mortality risk may contribute to the improvement of clinical management practices for these patients.

Low levels of hemoglobin (Hgb) are associated with adverse vascular outcomes in the general population,^2^ and CVD and anemia are important independent predictors for mortality.^3–5^

Anemia is frequently diagnosed in patients with CVD.^2,6,7^ Several studies have associated anemia with increased hospitalization and short-term mortality in patients with coronary disease.^8–11^ A retrospective study of 1498 Tunisian individuals with acute coronary syndrome found that the in-hospital mortality rate among patients with severe anemia is 22.1%.^9^ Meanwhile, a recent United States study has shown that the unadjusted hospital mortality rate among 9644 individuals in a cardiac intensive care unit is almost doubled in those with anemia compared with those without this condition.^11^ However, data regarding the long-term survival of patients with CVD and anemia are limited and inconsistent.^2,12^

A few longitudinal studies have associated anemia with poor long-term outcomes.^2,13^ Shu et al. reported that anemia is a significant risk factor for decreased survival during 36 months of follow-up among 7466 Canadian individuals with acute coronary syndrome.^13^ By contrast, authors of the Second National Health and Nutrition Examination Survey Mortality Study noted no significant relationship between anemia and vascular mortality among 8896 American individuals after approximately 17 years of follow-up.^12^ Similarly, another large population-based study conducted in the United States found no difference in 1-year mortality among anemic individuals with coronary heart disease.^14^

CVD is the most common cause of death in the United Arab Emirates (UAE), constituting 40% of total deaths in 2016,^15^ which is higher than the global average of 32%.^1^ Anemia is also prevalent in the UAE, affecting approximately 18% of women of reproductive age.^16^

The long-term survival of CVD patients with concomitant anemia in the UAE has not yet been studied. Determining the prognostic significance of anemia in CVD patients is essential to provide effective CVD risk assessment and management in high-risk patients. This study aimed to determine the long-term impact of anemia on mortality in UAE nationals with CVD over 10 years.

## Methods

### Study setting and participants

This retrospective cohort study was conducted in specialty and sub-specialty outpatient clinics at Tawam Hospital, a large tertiary healthcare government facility in Al-Ain City, UAE. Patients were recruited from 1 April 2008 to 31 December 2008 and included in subsequent follow-up until 31 December 2019. Their data were obtained retrospectively from the hospital electronic medical record (EMR) management system. The EMR database includes sociodemographic information, clinical and biochemical data, medication usage, and death record information. Ethics approval was obtained from Tawam Hospital's Medical Research and Ethics Board (MF2058-2020-720). The requirement for informed consent was waived because patient records and information were anonymized and de-identified before analysis.

We included UAE nationals aged 18 years or older with a documented baseline history of CVD (defined as a history of either heart disease [a documented diagnosis of unstable angina, stable angina, myocardial infarction, coronary artery bypass graft surgery, percutaneous coronary intervention, atrial fibrillation, or congestive heart failure], cerebrovascular disease [a documented diagnosis of stroke or transient ischemic attack], or peripheral vascular disease [a documented diagnosis of peripheral artery disease]). Patient data were reviewed from the time of recruitment until death or until the end of the study period (31 December 2019), whichever occurred first.

### Variables and definitions

Baseline variables retrieved from the EMR included sociodemographic information, including age, sex, and smoking history; clinical and biochemical data, including body mass index (BMI), systolic and diastolic blood pressure, serum Hgb levels, fasting serum lipid profile, estimated glomerular filtration rate (eGFR), history of diabetes, and malignancy; and medication usage.

Obesity was defined as BMI of ≥ 30 kg/m^2^.^17^ Hypertension (HTN) was defined as systolic blood pressure of ≥ 140 mmHg or diastolic blood pressure of ≥ 90 mmHg, or receipt of pharmacological treatment for HTN.^18^ Anemia was defined as Hgb of < 130 g/L in men and < 120 g/L in women.^19^ Total Hgb levels were measured using an automated hematology analyzer and colorimetric methods. Dyslipidemia was defined as serum high-density lipoprotein level of < 1.03 mmol/L, serum triglyceride level of ≥ 2.26 mmol/L, or receipt of lipid-lowering therapy.^20^ Patients with eGFR of < 60 mL/min/1.73 m^2^ were defined as having chronic kidney disease (CKD). The Chronic Kidney Disease Epidemiology Collaboration equation based on serum creatinine was used to calculate the eGFR.^21^ History of diabetes was determined based on an established diagnosis by a physician. Malignancy was defined as a definitive diagnosis of any type of cancer. Smoking status was described as a previous or current history of smoking tobacco products. Antithrombotic drugs included any antiplatelet agents and any anticoagulant drugs.

The main outcome of interest in this study was all-cause mortality, determined as death from any cause. CVD mortality was defined as sudden death or death as a result of a fatal myocardial infarction or fatal stroke. All deaths were confirmed by a careful review of death certificates and the EMR.

### Statistical analyses

Prior to analyses, patients were categorized based on their anemia status. The baseline variables were tested for association using the independent-samples t-test and Fisher's exact test (two-tailed) for continuous and categorical variables, respectively, among the groups. Univariate Kaplan–Meier analyses were conducted to measure the survival functions for all-cause mortality, and log-rank tests were used for comparisons between the groups. A Cox regression model was used to evaluate the association between anemia (categorical) and all-cause mortality while adjusting for other variables (age, sex, history of diabetes, HTN, dyslipidemia, CKD, malignancy, smoking, use of antithrombotic drugs, and obesity). The proportional hazards assumption was assessed using log–log plots. The results were expressed as hazard ratios (HRs) and 95% confidence intervals (CIs). All statistical analyses were performed using R software v3.6.3 (R Foundation for Statistical Computing, Vienna, Austria) and SPSS Statistics for Windows v26 (IBM Corp, Armonk, NY, USA). Two-tailed p-values < 0.05 were considered statistically significant.

## Results

The study population initially included 243 UAE nationals aged 18 years or older. We excluded 19 patients, 18 of whom had missing Hgb data, and one of whom was lost to follow-up. A total of 224 (174 male and 50 female) individuals were included in the follow-up over a median period of 10.5 years (interquartile range [IQR] = 8.8–11.2 years). Table 1 shows the baseline characteristics classified by anemia status. The mean age of all individuals was 65.5 ± 11.6 years. Heart disease, cerebrovascular disease, and peripheral vascular disease were diagnosed in 69.2%, 38.8%, and 7.1% of all patients, respectively. A considerable number of individuals had HTN (93.3%) and/or dyslipidemia (95.1%), whereas more than half had diabetes (61.6%). Almost three-quarters of patients were receiving antithrombotic drugs. At baseline, the overall mean Hgb level was 124.9 ± 27.8 g/L; 46% of all individuals had anemia with a mean Hgb level of 105.5 ± 28.0 g/L. Individuals with anemia tended to be older (68 vs. 63 years, p = 0.001) and were more likely to have a history of CKD (37.5% vs. 17.5%, p = 0.001) compared with those without anemia at baseline.

**Figure 1.**
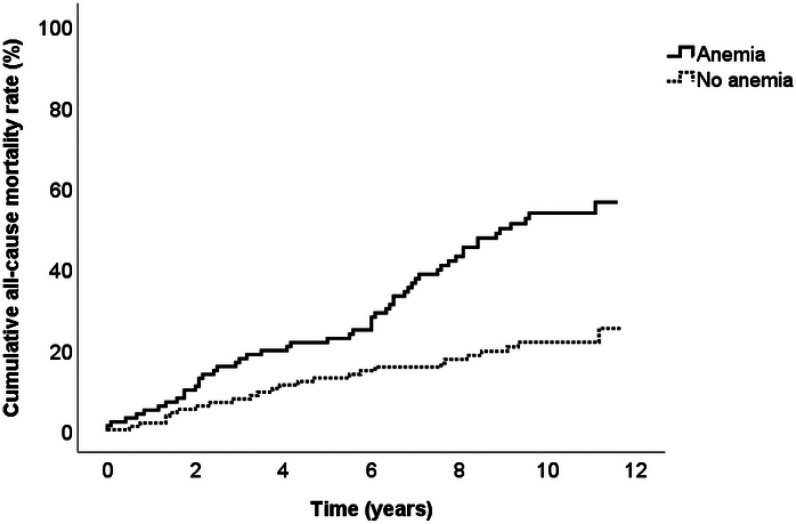
Unadjusted Kaplan–Meier one minus survival curves showing the cumulative all-cause mortality rate among United Arab Emirates nationals with cardiovascular disease, stratified according to the presence or absence of anemia (log-rank of < 0.001).

**Figure 2.**
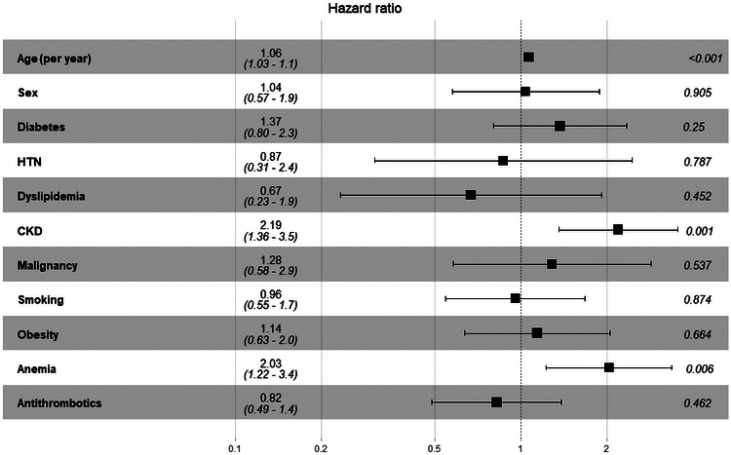
Adjusted^a^ hazard ratio (95% confidence interval) of predictors associated with all-cause mortality in patients with cardiovascular disease (n = 224). HTN, hypertension; CKD, chronic kidney disease.^a^All covariates in the figure were included in the multiple Cox regression analysis.

At the end of the follow-up period, 77 (34.4%) deaths had occurred, of which 34 (44.2%) were due to CVD. Half of the individuals with anemia died during follow-up (n = 52) compared with 20.8% of those without anemia (n = 25). During the 10 years of follow-up, the all-cause mortality rate in individuals with anemia was statistically significantly higher than that in individuals without anemia (55% vs. 20%; HR = 2.03, 95% CI = 1.22–3.40, p = 0.006) (Figures 1 and 2). In addition to the presence of anemia, multivariable Cox regression analysis showed that age and CKD were statistically significant predictors of death (HR = 1.06, 95% CI = 1.03–1.10, p < 0.001 and HR = 2.19, 95% CI 1.36–3.50, p = 0.001, respectively).

## Discussion

To the best of our knowledge, this study is the first to assess the long-term risk of mortality in CVD patients with and without anemia in the UAE. In our study, nearly half of the patients with CVD had anemia. Several studies have reported the prevalence of anemia in the context of vascular disease, with rates ranging from 27% to 43%.^22–25^ Variation in estimates of prevalence can be attributed to the characteristics of the populations being studied. For instance, the prevalence of anemia is considerably higher in individuals from the Middle East compared with Western populations because of the increased rates of thalassemia, hemoglobinopathies, and nutritional deficiencies.^26^ Furthermore, the majority of cases of anemia in the adult Arab population are caused by iron deficiency;^9^ nutritional education and health promotion programs are therefore necessary to lower the prevalence of anemia among high-risk individuals in the UAE and potentially improve clinical outcomes.^27,28^

Our findings demonstrated that the survival rates of individuals with CVD with and without anemia differed significantly (Figures 1 and 2). Several previous studies have investigated and validated the association between baseline anemia and mortality in individuals with CVD.^8,24,29,30^ Although these study populations were not comparable to the UAE population, they had similar results. One study from the Middle East showed that hospitalized patients with anemia and acute coronary syndrome had 1.34-fold and 1.22-fold increases in mortality at one month and one year, respectively.^24^ Another study that recruited individuals from the Myocardial Infarction Registry in Germany found that anemia is associated with a significant increase in long-term all-cause mortality among patients with acute myocardial infarction (AMI).^30^ However, this conclusion was based on patients with AMI only and not those with CVD in general. A recently published systematic review and meta-analysis on the prognostic impact of anemia in patients with heart failure reported an estimated pooled mortality odds ratio (OR) of 1.43 (95% CI = 1.25–1.63, p < 0.001) in those with anemia.^31^ Moreover, the OR was calculated from 10 studies in which outcomes were reported for only one year. A direct comparison between these studies and ours may not be possible because of variations in inclusion and exclusion criteria and the duration of the follow-up period among the studies.

Several mechanisms may explain the increased mortality risk observed in patients with anemia and CVD. For instance, the short-term effects of increased cardiac output with anemia result from decreasing afterload and increasing preload.^32^ Over time, the presence of chronic anemia may lead to left ventricular hypertrophy, which is considered a poor prognostic factor.^33^ Therefore, anemia may worsen vascular ischemia in susceptible individuals and increase their risk of mortality.

In our study, the presence of CKD was found to be a statistically significant predictor of all-cause mortality. Reduced renal function affects the survival of patients with pre-existing vascular disease and anemia.^34,35^ The combination of anemia, CVD, and CKD is referred to as cardiorenal anemia syndrome.^36^ Each component is believed to independently affect the other components of the syndrome, increasing the risk of mortality.^35^

Anemia is common in older individuals, with an estimated prevalence of 17% in the general elderly population.^37^ In addition, age plays a key part in the decline of cardiovascular function, resulting in an increased risk of mortality in older adults,^38,39^ which is consistent with the results of our study.

Sex-based studies have reported a positive association between male sex and death in patients with existing CVD.^38,40,41^ However, in the present study, this association was not statistically significant. This finding may be attributed to the smaller relative sample size of our study compared with previous studies.

## Strengths and limitations

Our study was conducted in a population of UAE nationals with CVD over a follow-up period of 10 years. Assessment of the long-term association between anemia and adverse clinical outcomes over an extended follow-up period improves reliability. However, our study has some limitations. First, this study was conducted in a single center; thus, the generalizability of the findings to the general UAE national population needs to be considered with caution. Second, selection bias may have occurred because of the retrospective nature of the study. Third, although the study was adjusted for differences in the baseline factors, other unmeasured confounding variables associated with anemia may have influenced clinical outcomes. Finally, data on the changes in Hgb concentration during the follow-up period were not available, which could have affected the results.

## Conclusion

This study demonstrated that anemia is a strong independent risk factor for long-term all-cause mortality in UAE nationals with a history of CVD. Our findings suggest the need to stratify patients with CVD according to their anemia status and encourage early intervention and treatment by promoting nutritional education programs. Future prospective studies should investigate targeted therapeutic strategies in this population.

## Declarations

### Ethics approval and consent to participate

This study was approved by the Research Ethics Committee of Tawam Hospital (MF2058-2020-720). The requirement for informed consent was waived because patient records and information were anonymized and de-identified before analysis.

### Availability of data and materials

Data supporting the findings of this study are available from the corresponding author upon reasonable request.

### Competing interests

The authors declare no conflicts of interest with respect to the authorship and/or publication of this article.

### Funding

The authors received no financial support for the research, authorship, or publication of this article.

## Figures and Tables

**Table 1:** Baseline characteristics of the study cohort stratified by anemia status.

Characteristic	Total (*n* = 224)	No anemia (*n* = 120)	Anemia (*n* = 104)	p-value

**Age (years), mean ± SD**	65.5 ± 11.6	63.1 ± 12.5	68.3 ± 9.7	0.001

**Men, n (%)**	174 (77.7)	95 (79.2)	79 (76.0)	0.630

**Diabetes mellitus, n (%)**	138 (61.6)	70 (58.3)	68 (65.4)	0.335

**HTN, n (%)**	209 (93.3)	112 (93.3)	97 (93.3)	1.000

**Dyslipidemia, n (%)**	213 (95.1)	113 (94.2)	100 (96.2)	0.551

**CKD, n (%)**	60 (26.8)	21 (17.5)	39 (37.5)	0.001

**Malignancy, n (%)**	17 (7.6)	9 (7.5)	8 (7.7)	1.000

**Smoking, n (%)**	59 (26.3)	36 (30.0)	23 (22.1)	0.224

**Obesity, n (%)**	63 (28.1)	39 (32.5)	24 (23.1)	0.137

**Anemia, n (%)**	104 (46.4)			

**Antithrombotic drugs, n (%)**	162 (72.3)	91 (75.8)	71 (68.3)	0.232

**Hgb (g/L), mean ± SD**	124.9 ± 27.8	141.7 ± 12.4	105.5 ± 28.0	< 0.001

**Mortality outcomes, n (%)**				

**CVD mortality**	34 (15.2)	13 (10.8)	21 (20.2)	

**All-cause mortality**	77 (34.4)	25 (20.8)	52 (50.0)	


SD, standard deviation; HTN, hypertension; CKD, chronic kidney disease; Hgb, hemoglobin; CVD, cardiovascular disease.
